# The risk of all-cause mortality associated with anxiety: a retrospective cohort study using ‘The Health Improvement Network’ database

**DOI:** 10.1186/s12888-023-04877-8

**Published:** 2023-06-05

**Authors:** Rebecca Russell, Sonica Minhas, Joht Singh Chandan, Anuradhaa Subramanian, Noel McCarthy, Krishnarajah Nirantharakumar

**Affiliations:** 1 Institute of Applied Health Research, College of Medical and Dental Sciences, University of Birmingham, Birmingham, B152TT UK; 2 Trinity College Dublin, Dublin, Ireland

**Keywords:** General Practice, Anxiety disorders, Mortality, Outcome studies, Epidemiology

## Abstract

**Background:**

Anxiety is a prevalent condition with a substantial associated burden of morbidity. Previous literature investigating effects of anxiety on mortality rates has found conflicting results. This is in part due to inadequate consideration of comorbid depression as a confounder and analysing sub-types of anxiety together. The objective of this study was to compare mortality risks in people diagnosed with anxiety.

**Methods:**

We undertook a retrospective cohort study using the ‘The Health Improvement Network’ database (a UK primary care dataset) between 1st January 2005 to 1st January 2018. 345 903 patients with anxiety (exposed group) were matched to 691 449 unexposed patients. Cox regression analyses were used to adjusted hazard ratios (HRs) for mortality risk.

**Results:**

During the study period 18 962 patients (5·5%) died in the exposed group compared to 32 288 (4·7%) in the unexposed group. This translated into a crude HR for all of 1·14 (95% CI 1·12 − 1·16), which remained significant after adjustment for key co-variates (including depression) giving a final HR of 1·05 (95% CI 1·03 − 1·07). When broken down by sub-type of anxiety (10·3% (35, 581) had phobias, 82·7% (385,882) has ‘other’ types, and 7·0% (24,262) had stress related anxiety) there were markedly different effect sizes. The adjusted model for the stress-related anxiety sub-type demonstrated a HR of 0·88 (95% CI 0·80 − 0·97). Conversely, the HR was increased in ‘other’ sub-types to 1·07 (95% CI 1·05 − 1·09) and non-significant in phobia types of anxiety.

**Conclusion:**

A complex relationship is found between anxiety and mortality. The presence of anxiety slightly increased the risk of death, but this risk varies depending on the type of anxiety diagnosed.

**Supplementary Information:**

The online version contains supplementary material available at 10.1186/s12888-023-04877-8.

## Introduction

Anxiety encompasses a heterogeneous group of mental illnesses, often managed in primary care. A 2013 UK survey found the weekly prevalence of anxiety was 6.6% and a 2020 study using UK Biobank data found the lifetime prevalence of anxiety was 7% [1, 2]. There are several sub-types of anxiety, with International Classification of Disease criteria (ICD-10) broadly classifying the most common sub-types as: F40 phobias, F41 “other” types (including generalised anxiety disorder [GAD] and panic), F42 obsessive-compulsive disorder (OCD), and F43 stress-related (including post-traumatic stress disorder [PTSD]) [3]. GAD is the most frequently reported mental health disorder in the UK [4].

The mortality and morbidity effects of anxiety are profound and it has been described that the population attributable risk for excess mortality due to anxiety is 4.3%, higher than even that of psychosis (0.63%) [5]. Whereas, while the relationship between severe mental illness (SMI) and increased mortality rates (MR)/ mortality rate ratio (MRR) is well documented and showing a consistent relationship, the association between anxiety and mortality is less well studied and the effect size reported appears inconsistent [6–14]. It has been previously noted that large studies are required to appropriately power any future investigation examining this relationship [6].

A systematic review found the pooled relative risk for mortality attributable to anxiety to be 1·43 (95% CI 1·24 − 1·64), however, this was limited as it lacked control for important confounders such as comorbid mental ill health [5]. An alternative systematic review including prospective cohort studies which also was able to control for comorbid depression found the overall HR of mortality in patients diagnosed with anxiety when compared to controls to be non-significant (HR 1·01, 95% CI 0·96 − 1·06) [12]. One UK study investigated the effects of questionnaire validated anxiety and mortality and found a non-significant effect with a HR of 1·05 (95% CI 0·96 − 1·14) [10]. Similarly, anxiety disorders were not found to have any excess mortality risk after adjusting for comorbid depression in another European study (Finnish population) [11]. A previous review has suggested that the null results may have been due to the different sub-types of anxiety being studied together [12]. The only study the authors were able to identify which clearly delineated risk between different sub-types of anxiety included data taken from a Danish patient registry [6]. In this study, MRRs for different anxiety sub-types (acute stress reaction, agoraphobia, GAD, panic disorder, post-traumatic stress disorder, social phobia and specific phobias) were significantly increased with estimates varying from 1·31 to 1·69 (dependent on type of anxiety) illustrating a differential effect [6]. However, the findings from this study may not be generalisable to the risks associated to anxiety which presents in primary care as this dataset was largely derived from secondary care hospital and in-patient psychiatry attendances. Of note, another more recent UK study using a British Birth cohort attempted to assess how affective symptoms (including anxiety) over the life course relates with all-cause mortality risk [15]. Of note, this study examined other potential confounders which may play a role in this relationship (beyond comorbid depression), such as but not limited to wider comorbidities, anxiolytic use, smoking, diet among other factors [15]. Such broader confounders have also been acknowledged as potential confounders in the relationship between anxiety and mortality and when adjusted for in global settings generally reduces the effect size describing the risk of mortality in patients with anxiety [16].

To date, to the authors knowledge there has not been a UK-based retrospective cohort study based in primary care settings investigating the mortality risks associated with an anxiety diagnosis, whilst considering the effect of both comorbid depression and sub-types of anxiety. This is important to clarify as the largest burden of anxiety occurs in a primary care setting, and the risks associated with the disease are important to guide physical health support that may be required.

## Methods

### Study design, population and data source

A retrospective open cohort study, utilising quantitative data from ‘The Health Improvement Network’ (THIN) database, was conducted comparing patients with coded anxiety diagnoses (exposed group) to unexposed patients. Patients who were over the age of 18 years were eligible for cohort entry, and the study period was set between 1st January 2005 to 1st January 2018.

THIN database contains anonymised electronic records taken from over 700 general practices using Vision software [17]. Patients in the dataset are deemed to be representative of the general population [18]. Data relating to symptoms, examinations and diagnoses are recorded using a hierarchical clinical coding system called Read codes [19].

To mitigate the risk of under-recording of the outcome of interest and ensure data quality, GPs were only included from the later of the following dates: 1 year after installation of Vision software systems or acceptable mortality recording date (the date, or patient registration date into the practice [20]. Data extraction was facilitated using DExtER, a software tool designed to support the extraction, transformation and loading of datasets for epidemiological purposes [21]. To mitigate the risk of under-recording of the outcome of interest and ensure data qualityGPs from the later of the following dates: 1 year after or.

### Exposure and outcome definition

The exposure was a Read code recorded diagnosis of any incident anxiety disorder (meaning the anxiety diagnosis was given during the study period). Read codes that may indicate anxiety diagnoses were assessed for their suitability and sub-typed according to the ICD-10 [3] criteria by the author who is a qualified psychiatrist and has been previously used in research [22–28]. Coding for anxiety has been previously validated in UK primary care data [29–31]. The outcome measure was all-cause mortality.

Read code lists can be found from Additional file 1.

### Selection of unexposed group and follow up period

Exposed patients were matched by age (± one year) and gender to two unexposed participants from the same general practice (GP) who did not have a recorded diagnosis of anxiety. This selection formed the primary cohort.

The index date (when follow-up measurement began) was defined as a year after the anxiety disorder was diagnosed in the exposed group. This was done to exclude end-of-life anxiety diagnoses where there may be issues with reverse causality i.e. known expected deaths causing anxiety in the patient. For the unexposed group the corresponding exposure patient’s index date was assigned to avoid immortality time bias [32]. The patients were then followed-up until the exit date, defined as the earliest of the following censoring events: patient transferred out of the practice, patient death (outcome of interest), practice leaves the Vision system/THIN database or the study end date.

### Co-variates

The following co-variates at baseline were considering in our modelling due to their independent association with mortality: age, gender, ethnicity, alcohol use, smoking status, Charlson Comorbidity Index (CCI) [33], Townsend deprivation index [34], depression or other SMI. The absence of a smoking status was taken to indicate non-smoking status, as has been found consistently in previous primary care research [35]. For Townsend deprivation quintiles, ethnicity and drinking status a missing category was used in the analysis.

### Data analysis

STATA version SE 15 (Statacorp 2017) was used for the analysis, with statistical significance set at *p* < 0.05 (two-sided).

Cox proportional hazard models (assumptions were tested and no violations noted) were used to calculate crude HRs and adjusted HRs. These models were constructed in a sequence of pre-defined steps, which had been selected based on their known importance from previous work and as such deemed both clinically important and explanatory.

The steps in adjustment were: (1) matched demographic factors (age, gender) (2) other demographic factors (ethnicity, deprivation [as measured by Townsend deprivation quintiles] (3) behavioural factors (alcohol use, smoking status) (4) comorbid mental illness (depression, SMI), and (5) physical comorbidity (as measured by the CCI). Sensitivity analyses were performed for each sub-type of anxiety with their respective controls in the same steps.

Individual covariates effects were considered in the reported model, which did not include ethnicity as over half of the data were missing for this variable. The pairwise analyses for the anxiety sub-types were limited to those whose numbers accounted for greater than 1% of the database.

## Results

A total of 345 903 patients had a coded incident anxiety diagnoses during the study period who were matched by age and gender to 691 449 unexposed patients. The total study time for all the participants was 5 528 153 person years. A greater proportion of the exposed group were more deprived, current smokers, heavy drinker and had a high proportion of comorbid mental and physical illness. More details can be seen in Table 1. The subtypes of anxiety were distributed as: 10·3% (35, 581) phobias, 82·7% (385,882) ‘other’ types, and 7·0% (24,262) stress related. The remaining sub-types accounted for less than 1% of all anxiety cases, and so were not considered in the sub-type analysis.

**Table 1:** Baseline characteristics of participants

Characteristics	Unexposed group	Exposed group
**Total cohort**	691 449	345 903
**Number of deaths**	32 288 (4·7)	18 962 (5·5)
**Follow-up, median (IQR) years**	4·3 (1·7–8·1)	4·5 (1·9 − 8·4)
**Age, median (IQR) years**	43·0 (31·5–56·4)	43·0 (31·5–56·4)
**Male**	245 919 (35·6)	122 980 (35·6)
**Ethnic group**		
** Black**	7 570 (1·1)	2 218 (0·6)
** Mixed race**	2 018 (0·3)	979 (0·3)
** White**	247 556 (35·8)	137 640 (39·6)
** Other**	5 107 (0·7)	1 381 (0·4)
** South Asian**	11 884 (1·7)	4 087 (1·2)
** Missing**	417 314 (60·4)	200 154 (57·9)
**Townsend deprivation quintile**		
** 1 (least deprived)**	154 245 (22·3)	69 425 (20·1)
** 2**	130 804 (18·9)	61 111 (17·7)
** 3**	126 405 (18·3)	63 231 (18·3)
** 4**	110 137 (15·9)	59 887 (17·3)
** 5 (most deprived)**	80 121 (11·6)	47 240 (13·7)
**Missing**	89 737 (13·0)	45 009 (13·0)
**Current smoker**	141 762 (20·5)	98 779 (28·6)
**Alcohol use**		
** Non-drinker**	107 507 (15·6)	57 456 (16·6)
** Light-moderate drinker**	420 498 (60·8)	210 698 (60·9)
** Heavy drinker**	19 434 (2·8)	23 458 (6·8)
** Missing**	144 010 (20·8)	54 291 (15·7)
**Comorbid depression**	72 824 (10·5)	171 294 (49·5)
**Comorbid SMI**	5 139 (0·7)	6 796 (2·0)
**Charlson comorbidity index**		
** 0 (no comorbidities)**	518 737 (75·0)	239 378 (69·2)
** 1**	123 192 (17·8)	75 709 (21·9)
** 2**	32 699 (4·7)	19 605 (5·7)
** 3**	10 582 (1·5)	6 990 (2·0)
** 4**	3 428 (0·5)	2 222 (0·6)
** ≥5**	2 811 (0·4)	1 999 (0·6)

Numbers are counts (percentages) unless otherwise stated

The Mann-Whitney *U-*test was conducted for continuous parameters and *Χ*^*2*^ for categorical variables

*SMI* Severe mental illness, *IQR* Interquartile range

**Fig. 1:**
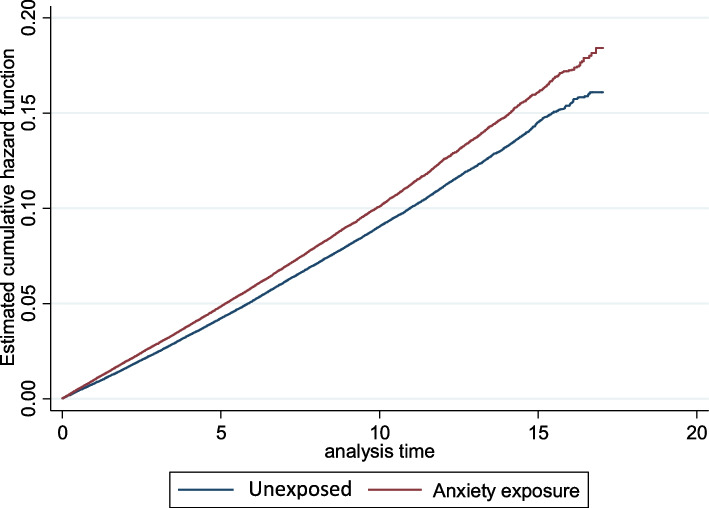
Nelson-Aalen cumulative hazard function associated with unexposed and exposed patients (Analysis time in years)

There were 32 288 deaths in the exposed group compared to 18 962 in the exposed group. Taking into consideration person years follow, this related to a crude mortality rate in the unexposed group was 8·9 per 1 000 person years compared to 10·1 per 1 000 person years in the exposed group. This translated into a crude HR of 1·14 (95% CI 1·12 − 1·16) depicted in Fig. 1; Table 2.

**Table 2:** Hazard ratios (HR) for death in participants with a diagnosis of anxiety

Model	HR (95% CI)
**Crude HR**	1·14 (1·12 − 1·16)
**Adjusted: model 1**	1·20 (1·17 − 1·22)
**Adjusted: model 2**	1·19 (1·17 − 1·21)
**Adjusted: model 3**	1·14 (1·12 − 1·17)
**Adjusted: model 4**	1·07 (1·05 − 1·09)
**Adjusted: model 5**	1·05 (1·03 − 1·07)

HR adjusted for *model 1*: age and sex;

*model 2*: age, sex, ethnicity and Townsend deprivation quintile;

*model 3*: age, sex, ethnicity, Townsend deprivation quintile, alcohol and smoking use;

*model 4*: age, sex, ethnicity, Townsend deprivation quintile, alcohol use, smoking use, comorbid depression and SMI;

*model 5*: age, sex, ethnicity, Townsend deprivation quintile, alcohol use, smoking use, comorbid depression and SMI, CCI

Dependent on the adjustment model used, the analysis showed an excess mortality of between 5 and 20%. The largest drop in risk occurred after depression and SMI were included in the model. See Table 2 for further details. The fifth model demonstrating an adjusted HR of 1·05 (95% CI 1·03 − 1·07) is shown in further detail in Table 3. All of the covariates had larger effects on the mortality rate than anxiety alone. 

The raised mortality associated with anxiety may not be as substantial as that associated with some other mental ill health conditions, due to the prevalence of anxiety, this still translates into a substantial preventable public health burden. Further research should aim to examine bio-psycho-social pathways responsible for the inflated mortality rates to identify any suitable targets for interventions which may include more stringent monitoring of physical health and lifestyle choices made by patients diagnosed with anxiety.

**Table 3:** Full model for adjusted: model 5

Covariate	HR (95% CI)
**Anxiety**	1·05 (1·03 − 1·07)
**Age**	1·10 (1·10 − 1·10)
**Sex** ^**a**^	0·73 (0·72 − 0·74)
**Townsend deprivation quintile** ^**b**^	
** 2**	1·13 (1·10 − 1·16)
** 3**	1·19 (1·15 − 1·22)
** 4**	1·35 (1·32 − 1·39)
** 5**	1·46 (1·42 − 1·51)
** Missing**	1·17 (1·13 − 1·21)
**Use of alcohol** ^**c**^	
** Light-moderate drinker**	0·83 (0·81 − 0·85)
** Heavy drinker**	1·76 (1·69 − 1·82)
** Missing**	1·12 (1·09 − 1·16)
**Current smoking** ^**d**^	1·85 (1·81 − 1·89)
**Comorbid depression** ^**e**^	1·09 (1·07 − 1·12)
**Comorbid SMI** ^**f**^	1·48 (1·40 − 1·57)
**Charlson Index** ^**g**^	
** 1**	1·59 (1·55 − 1·62)
** 2**	2·06 (2·01–2·11)
** 3**	2·53 (2·45 − 2·62)
** 4**	3·18 (3·03–3·34)
** 5 or more**	5·06 (4·83 − 5·31)

Adjusted to include: age, sex, Townsend deprivation quintile, alcohol and smoking use, comorbid depression and severe mental illness, and Charlson comorbidity indicator·

*SMI* Severe mental illness

^a^ reference is male

^b^ reference is 1st deprivation quintile

^c^ reference is non-drinker

^d^ reference is non-smoker

^e^ reference is no depression

^f^ reference is no SMI

^g^ reference is Charlson Index Score of 0

When examining subtypes of anxiety, it appeared phobic anxieties showed no significant association with mortality, whereas stress-related conditions appeared to reduce mortality risk only after full adjustment with a HR of 0·89 (95% CI 0·80 − 0·98). ‘Other’ F41 diagnoses significantly raised the adjusted HR to 1·07 (95% CI 1·05 − 1·10) and remained significant throughout all of the models. See Table 4 for further details.

**Table 4:** Hazard ratios (HR) for death in participants with a diagnosis of phobic, ‘other’ or stress-related anxiety sub-types

Model	Phobia HR (95% CI)	Other anxiety HR (95% CI)	Stress-related HR (95% CI)
**Crude HR**	1·05 (0·98 − 1·12)	1·15 (1·13 − 1·18)	0·96 (0·87 − 1·06)
**Adjusted: model 1**	1·06 (0·99 − 1·13)	1·22 (1·18 − 1·24)	0·98 (0·89 − 1·08)
**Adjusted: model 2**	1·04 (0·98 − 1·12)	1·21 (1·19 − 1·24)	0·97 (0·88 − 1·06)
**Adjusted: model 3**	1·02 (0·95 − 1·09)	1·17 (1·14 − 1·19)	0·92 (0·84 − 1·02)
**Adjusted: model 4**	1·00 (0·93 − 1·07)	1·09 (1·07 − 1·12)	0·89 (0·81 − 0·99)
**Adjusted: model 5**	0·98 (0·91 − 1·05)	1·07 (1·05 − 1·10)	0·89 (0·80 − 0·98)

HR adjusted for:

*model 1*: age and sex;

*model 2*: age, sex, ethnicity and Townsend deprivation quintile;

*model 3*: age, sex, ethnicity, Townsend deprivation quintile, alcohol and smoking use;

*model 4*: age, sex, ethnicity, Townsend deprivation quintile, alcohol and smoking use, comorbid depression and SMI;

*model 5*: age, sex, ethnicity, Townsend deprivation quintile, alcohol and smoking use, comorbid depression and SMI, and CCI

The clinically important model (model 5), excluding ethnicity, and its effects on all the covariates for each of the three anxiety sub-types is shown in Table 5. This shows similar effects of the covariates as found with the combined anxiety results, except being anxious is protective for stress-related anxieties but has no effect in phobias. Additionally, it appears the effect of the relationship between SMI and mortality varies as a confounder between the subtypes of anxiety. In stress-related anxiety, SMI has a much greater impact than in phobia or other anxiety types.

**Table 5:** Adjusted Hazard ratios (HR) for death in participants with a diagnosis of subtypes of anxiety using Cox proportional modelling

Covariate	Phobias HR (95% CI)	Other anxiety HR (95% CI)	Stress-related HR (95%CI)
**Anxiety**	0·98 (0·91 − 1·05)	1·07 (1·05 − 1·09)	0·88 (0·80 − 0·97)
**Age**	1·10 (1·09 − 1·10)	1·10 (1·10 − 1·10)	1·10 (1·10 − 1·10)
**Sex** ^**a**^	0·73 (0·68 − 0·78)	0·73 (0·72 − 0·75)	0·70 (0·64 − 0·77)
**Townsend deprivation quintile** ^**b**^			
** 2**	1·12 (1·10 − 1·36)	1·12 (1·08 − 1·15)	1·20 (1·04 − 1·38)
** 3**	1·25 (1·13 − 1·39)	1·19 (1·15 − 1·22)	1·09 (0·93 − 1·26)
** 4**	1·47 (1·32 − 1·63)	1·34 (1·30 − 1·38)	1·44 (1·24 − 1·67)
** 5**	1·53 (1·36 − 1·72)	1·45 (1·41 − 1·50)	1·54 (1·32 − 1·79)
** Missing**	1·34 (1·18 − 1·52)	1·15 (1·11 − 1·20)	1·19 (0·98 − 1·43)
**Use of alcohol** ^**c**^			
** Light-moderate**	0·77 (0·71 − 0·84)	0·84 (0·82 − 0·86)	0·85 (0·75 − 0·96)
** Heavy**	1·79 (1·56 − 2·06)	1·75 (1·68 − 1·83)	1·80 (1·50 − 2·17)
** Missing**	1·13 (1·02 − 1·27)	1·12 (1·09 − 1·16)	1·10 (0·92 − 1·30)
**Current smoking** ^**d**^	1·96 (1·82 − 2·12)	1·83 (1·79 − 1·88)	2·09 (1·88 − 2·32)
**Comorbid depression** ^**e**^	1·15 (1·06 − 1·26)	1·08 (1·05 − 1·10)	1·07 (0·96 − 1·21)
**Comorbid SMI** ^**f**^	1·62 (1·28 − 2·04)	1·44 (1·36 − 1·53)	2·87 (2·17 − 3·82)
**Charlson Index** ^**g**^			
** 1**	1·56 (1·44 − 1·69)	1·59 (1·55 − 1·63)	1·60 (1·43 − 1·80)
** 2**	2·21 (2·01–2·44)	2·04 (1·99 − 2·10)	2·09 (1·80 − 2·41)
** 3**	2·42 (2·10 − 2·78)	2·52 (2·43 − 2·61)	3·26 (2·71 − 3·92)
** 4**	3·91 (3·23 − 4·73)	3·11 (2·96 − 3·27)	4·04 (3·16 − 5·18)
** 5 or more**	7·42 (6·19 − 8·89)	4·88 (4·64 − 5·13)	7·41 (5·82 − 9·43)

Adjusted to include: age, sex, Townsend deprivation quintile, alcohol and smoking use, comorbid depression and SMI, and Charlson comorbidity indicator·

*SMI* Severe mental illness

^a^ reference is male

^b^ reference is 1st deprivation quintile

^c^ reference is non-drinker

^d^ reference is non-smoker

^e^ reference is no depression

^f^ reference is no SMI

^g^ reference is Charlson Index 0

## Discussion

### Summary

To the authors knowledge this was the first study investigating the mortality risks associated with an anxiety diagnosis, whilst considering the effect of both comorbid depression and sub-types in a UK primary care database. Our results showed a crude 14% increase in the risk of mortality when diagnosed with anxiety, dropping to a smaller but still significant increase of 5% after adjustment. For the sub-types, only the ‘other’ types of anxiety increased mortality risks, with a 7% increase following adjustment. There was evidence for stress-related conditions leading to a 12% reduction in risk at this level of adjustment. The substantial drop in risk when accounting for important covariates demonstrates that co-associated conditions play an important role in the relationship between anxiety and mortality.

### Strengths and limitations

The findings of this study should be considered in light of its limitations. Due to the retrospective nature of the study, the study is limited by the accuracy of contemporaneous recording. Regarding the sub-types, GAD and panic disorder (which would account for most of the “other” diagnoses) are thought to account for around 50% of all anxiety diagnoses, with phobias accounting for around 10%, and OCD accounting for around 1% [36]. Our data contained about 80% “other” types, with 10% phobias and less than 1% for OCD. Hence, the phobia data appears to be more reliable than that for the other sub-types. This may have occurred for several reasons: coding inaccuracy, diagnoses being more accurately sub-typed in secondary care, or the ‘other’ category being used secondarily to its rather inherent sounding generic nature. Hence, although the findings indicate that there may be differences between the sub-types that warrant further investigation, the diagnostic typing is probably not entirely clinically accurate according to ICD-10 criteria. However, it does reflect how coding occurs in primary care settings, and so still reflects the association with mortality of such diagnoses from these settings. Alternatively, the stress-related category lends itself to being used for many people who may present to primary care with “stress”, which need not necessarily reach the clinical threshold for an anxiety diagnosis, hence making this sub-type appear protective as it may be in part a non-clinical population.

### Comparison with existing literature

Prior to this study there have been several conflicting results in this area, with studies derived from secondary patients having had higher HRs than found (may be due to greater severity of symptomology or greater burden of SMI) or other cohorts showing insignificant association although may not have considered sub-types of anxiety [6–14].

Considering the effect of individual covariates in our modelling, the variables with the largest effects when considered on their own were increased severity of physical comorbidities, smoking status, severe mental illness, heavy alcohol use and being socioeconomically deprived. Comorbid depression and age also increased the mortality risks, though to a lesser extent. One study did find that HR increased for males only, which are reflected in our findings [9]. However, it is also possible that this may reflect other potential influences, including females seeking help for psychological problems at an earlier stage, or a propensity for professionals to diagnose psychological problems more readily in females [37].

The Danish registry study examining subtypes of anxiety found that phobias increase the mortality risk by 30–50%, “other” types by 30–60%, and stress-related conditions by 50–70% [6]. This does not reflect our findings, where stress-related conditions appeared to reduce all-cause mortality risk, phobias did not show a significant effect on mortality HR and the increase in “other” types was much more moderate than presented in the Danish registry study. This may be in part as the Danish registry study included secondary care patients, and so this population may have experienced more severe symptomology [6]. Our study’s finding appear plausible, in that phobias may tend to cause acute anxiety and biological reactions, rather than a chronic over-activation of such systems as may occur in the “other” conditions such as GAD, which may have a greater impact on patient health.

## Conclusion

In conclusion, anxiety disorders as a group have been found to slightly increase the risk of death compared to those without anxiety after adjustment for clinically relevant covariates. Some sub-types even appear to have protective effects, which is reassuring. However, even though the raised mortality associated with anxiety may not be as substantial as that associated with some other mental ill health conditions, due to the prevalence of anxiety, this still translates into a substantial preventable public health burden. Further research should aim to examine bio-psycho-social pathways responsible for the inflated mortality rates to identify any suitable targets for interventions which may include more stringent monitoring of physical health and lifestyle choices made by patients diagnosed with anxiety.

## Supplementary information

**Additional file 1. **Read codes.

## Data Availability

Data can be requested from the corresponding author following appropriate permissions from the data provider IQVIA.

## Abbreviations

CCI: Charlson Comorbidity Index
GAD: Generalized Anxiety Disorder
HR: Hazard Ratios
MR: Mortality Rate
MRR: Mortality Rate Ratio
SMI: Serious Mental Illness
