# Supplementary material for: The risk of all-cause mortality associated with anxiety: a retrospective cohort study using ‘The Health Improvement Network’ database
Source: BMC Psychiatry. 2023 Jun 5;23:400. doi: 10.1186/s12888-023-04877-8 (PMC10240780; doi:10.1186/s12888-023-04877-8)
Supplement: Supplementary file 1 — Additional file 1. Read codes. [file 12888_2023_4877_MOESM1_ESM.docx]

Additional file 1

# Read codes

| **Read Code** | **Description** | **Excluded/Subtype** | **Comments** |
| --- | --- | --- | --- |
| 1466 | H/O: anxiety state | F41 Other |  |
| 8CAZ000 | Patient given advice about management of anxiety | F41 Other |  |
| 8HHp.00 | Referral for guided self-help for anxiety | F41 Other |  |
| E200.00 | Anxiety states | F41 Other |  |
| E200000 | Anxiety state unspecified | F41 Other |  |
| E200200 | Generalised anxiety disorder | F41 Other |  |
| E200300 | Anxiety with depression | Comorbid depression |  |
| E200400 | Chronic anxiety | F41 Other |  |
| E200500 | Recurrent anxiety | F41 Other |  |
| E200z00 | Anxiety state NOS | F41 Other |  |
| E202.12 | Phobic anxiety | F40 Phobia |  |
| E292000 | Separation anxiety disorder | Excluded | Childhood disorder, not incident |
| E2D0.00 | Disturbance of anxiety and fearfulness childhood/adolescent | Excluded | Childhood disorder, not incident |
| E2D0z00 | Disturbance anxiety and fearfulness childhood/adolescent NOS | Excluded | Childhood disorder, not incident |
| Eu05400 | [X]Organic anxiety disorder | Organic |  |
| Eu34114 | [X]Persistant anxiety depression | Comorbid depression |  |
| Eu40.00 | [X]Phobic anxiety disorders | F40 Phobia |  |
| Eu40y00 | [X]Other phobic anxiety disorders | F40 Phobia |  |
| Eu40z00 | [X]Phobic anxiety disorder, unspecified | F40 Phobia |  |
| Eu41.00 | [X]Other anxiety disorders | F41 Other |  |
| Eu41000 | [X]Panic disorder [episodic paroxysmal anxiety] | F41 Other |  |
| Eu41100 | [X]Generalized anxiety disorder | F41 Other |  |
| Eu41111 | [X]Anxiety neurosis | F41 Other |  |
| Eu41112 | [X]Anxiety reaction | F41 Other |  |
| Eu41113 | [X]Anxiety state | F41 Other |  |
| Eu41200 | [X]Mixed anxiety and depressive disorder | Comorbid depression |  |
| Eu41211 | [X]Mild anxiety depression | Comorbid depression |  |
| Eu41300 | [X]Other mixed anxiety disorders | F41 Other |  |
| Eu41y00 | [X]Other specified anxiety disorders | F41 Other |  |
| Eu41y11 | [X]Anxiety hysteria | F41 Other |  |
| Eu41z00 | [X]Anxiety disorder, unspecified | F41 Other |  |
| Eu41z11 | [X]Anxiety NOS | F41 Other |  |
| Eu51511 | [X]Dream anxiety disorder | F41 Other |  |
| Eu93000 | [X]Separation anxiety disorder of childhood | Excluded | Childhood disorder, not incident |
| Eu93100 | [X]Phobic anxiety disorder of childhood | Excluded | Childhood disorder, not incident |
| Eu93200 | [X]Social anxiety disorder of childhood | Excluded | Childhood disorder, not incident |
| Z4L1.00 | Anxiety counselling | F41 Other |  |
| E200100 | panic disorder | F41 Other |  |
| E200111 | panic attack | F41 Other |  |
| E202.00 | phobic disorders | F40 Phobia |  |
| E202.11 | social phobic disorders | F41 Other |  |
| E202000 | phobia unspecified | F40 Phobia |  |
| E202100 | agoraphobia with panic attacks | F40 Phobia |  |
| E202200 | agoraphobia without mention of panic attacks | F40 Phobia |  |
| E202D00 | fear of death | F40 Phobia |  |
| E28..00 | acute reaction to stress | F43 Stress |  |
| E280.00 | acute panic state due to acute stress reaction | F43 Stress |  |
| E281.00 | acute fugue state due to acute stress reaction | F43 Stress |  |
| E282.00 | acute stupor state due to acute stress reaction | F43 Stress |  |
| E283.00 | other acute stress reactions | F43 Stress |  |
| E283z00 | other acute stress reaction nos | F43 Stress |  |
| E284.00 | stress reaction causing mixed disturbance of emotion/conduct | F43 Stress |  |
| E28z.00 | acute stress reaction nos | F43 Stress |  |
| Eu4..00 | [x]neurotic, stress - related and somoform disorders | F43 Stress |  |
| Eu40000 | [x]agoraphobia | F40 Phobia |  |
| Eu40011 | [x]agoraphobia without history of panic disorder | F40 Phobia |  |
| Eu40012 | [x]panic disorder with agoraphobia | F40 Phobia |  |
| Eu40100 | [x]social phobias | F40 Phobia |  |
| Eu40112 | social neurosis | F40 Phobia |  |
| Eu41011 | [x]panic attack | F41 Other |  |
| Eu41012 | [x]panic state | F41 Other |  |
| Eu42.11 | [x]anankastic neurosis | F42 OCD |  |
| Eu42.12 | [x]obsessive-compulsive neurosis | F42 OCD |  |
| Eu43.00 | [x]reaction to severe stress, and adjustment disorders | F43 Stress |  |
| Eu43000 | [x]acute stress reaction | F43 Stress |  |
| Eu43012 | [x]acute reaction to stress | F43 Stress |  |
| Eu43y00 | [x]other reactions to severe stress | F43 Stress |  |
| Eu43z00 | [x]reaction to severe stress, unspecified | F43 Stress |  |
| Z522600 | flooding - obsessional compulsive disorder | F42 OCD |  |
| 146G.00 | H/O: agoraphobia | F40 Phobia |  |
| 1B1H.11 | Fear | F40 Phobia |  |
| 1Bb..00 | Specific fear | F40 Phobia |  |
| E202300 | Social phobia, fear of eating in public | F40 Phobia |  |
| E202400 | Social phobia, fear of public speaking | F40 Phobia |  |
| E202500 | Social phobia, fear of public washing | F40 Phobia |  |
| E202600 | Acrophobia | F40 Phobia |  |
| E202700 | Animal phobia | F40 Phobia |  |
| E202800 | Claustrophobia | F40 Phobia |  |
| E202900 | Fear of crowds | F40 Phobia |  |
| E202A00 | Fear of flying | F40 Phobia |  |
| E202B00 | Cancer phobia | F40 Phobia |  |
| E202C00 | Dental phobia | F40 Phobia |  |
| E202E00 | Fear of pregnancy | F40 Phobia |  |
| E202z00 | Phobic disorder NOS | F40 Phobia |  |
| E227z11 | Fear of ejaculation | F40 Phobia |  |
| E28z.11 | Examination fear | F40 Phobia |  |
| E28z.12 | Flying phobia | F40 Phobia |  |
| E2D0100 | Childhood and adolescent fearfulness disturbance | Excluded | Childhood disorder, not incident |
| Eu22y11 | [X]Delusional dysmorphophobia | F45 Somatoform |  |
| Eu40111 | [X]Anthropophobia | F40 Phobia |  |
| Eu40200 | [X]Specific (isolated) phobias | F40 Phobia |  |
| Eu40211 | [X]Acrophobia | F40 Phobia |  |
| Eu40212 | [X]Animal phobias | F40 Phobia |  |
| Eu40213 | [X]Claustrophobia | F40 Phobia |  |
| Eu40214 | [X]Simple phobia | F40 Phobia |  |
| Eu40300 | [X]Needle phobia | F40 Phobia |  |
| Eu40z11 | [X]Phobia NOS | F40 Phobia |  |
| Eu40z12 | [X]Phobic state NOS | F40 Phobia |  |
| Eu45212 | [X]Dysmorphophobia nondelusional | F40 Phobia |  |
| Eu45215 | [X]Nosophobia | F40 Phobia |  |
| Z481.00 | Phobia counselling | F40 Phobia |  |
| Z522400 | Desensitisation - phobia | F40 Phobia |  |
| Z522700 | Flooding - agoraphobia | F40 Phobia |  |
| E2...00 | Neurotic, personality and other nonpsychotic disorders | F41 Other |  |
| E20..00 | Neurotic disorders | F41 Other |  |
| E204.00 | Neurotic depression reactive type | Comorbid depression |  |
| E20y.00 | Other neurotic disorders | F41 Other |  |
| E20y200 | Other occupational neurosis | F41 Other |  |
| E20y300 | Psychasthenic neurosis | F41 Other |  |
| E20yz00 | Other neurotic disorder NOS | F41 Other |  |
| E20z.00 | Neurotic disorder NOS | F41 Other |  |
| E20z.11 | Nervous breakdown | F41 Other |  |
| E21..11 | Neurotic personality disorder | Excluded | Personality disorder, not incident |
| E210.00 | Paranoid personality disorder | Excluded | Personality disorder, not incident |
| E283100 | Acute posttrauma stress state | F43 Stress |  |
| E28z.13 | Stage fright | F40 Phobia |  |
| Eu34111 | [X]Depressive neurosis | Comorbid depression |  |
| Eu34113 | [X]Neurotic depression | Comorbid depression |  |
| Eu60000 | [X]Paranoid personality disorder | Excluded | Personality disorder, not incident |
| Eu60011 | [X]Expansive paranoid personality disorder | Excluded | Personality disorder, not incident |
| Eu60600 | [X]Anxious [avoidant] personality disorder | Excluded | Personality disorder, not incident |
